# Polyethylene-Grafted Gold and Silver Nanoparticles Using Catalyzed Chain Growth (CCG)

**DOI:** 10.3390/polym10040407

**Published:** 2018-04-06

**Authors:** Jannik Wagner, Wentao Peng, Philipp Vana

**Affiliations:** Institute of Physical Chemistry, Georg-August-University Göttingen, Tammannstr. 6, D-37077 Göttingen, Germany; jannik.wagner@stud.uni-goettingen.de (J.W.); wentaopengcustom@gmail.com (W.P.)

**Keywords:** polyethylene, modified gold nanoparticles, modified silver nanoparticles, thermoresponsive nanoparticles

## Abstract

We report an efficient synthesis route for the formation of gold/silver-core–PE-shell nanohybrids in a simple self-assembly approach using PE with strong aurophilicity and argentophilicity, via thiol- and trithiocarbonate terminated moieties. This united the unique properties of polyethylene (PE) with gold and silver nanoparticles, using the well-defined end-group design of PE. These nanocomposites showed a similar solubility as PE, as confirmed by dynamic light scattering, and could be fully incorporated into a polyethylene matrix with different particle contents, as visualized by transmission electron microscopy. Using UV/vis-spectroscopy, we observed reversible, thermoresponsive aggregation/deaggregation properties in the nanohybrids, validating the strong and effective anchoring of PE on gold/silver surfaces.

## 1. Introduction

Nanoscience and nanotechnology, including various types of nanoparticles and polymer matrices, have become prominent fields in research [1,2]. In recent years, gold and silver nanoparticles have gained increasing importance and popularity in functional material science, as well as in industrial applications. Beyond their organometallic catalytic activity, unique properties from their nano-scaled structure open up a wide range of applications. For instance, gold nanocomposites show high performance and flexibility in biosensoring due to their specific plasmonic optical properties [3,4]. Silver nanoparticles exhibit antimicrobial behavior and therefore have been widely employed in food packing and the biomedical industry [5,6]. The usage of gold or silver nanoparticles has been greatly enhanced with the introduction of polymer chemistry to form hybrid nanostructures and composite materials [7]. These nanocomposites complement the characteristics of functional polymers and thus provide improved mechanical, optical, or electric properties [8]. Additionally, a simple but important advantage of polymer capped nanocomposites is their high stability, as well as adjustable dispersibility into different environments, e.g., solvents or polymer matrices [9]. One of the most convenient techniques to modify a surface with polymers is the grafting-to approach. In this approach, pre-synthesized polymers with defined structures and functional anchoring groups can be grafted onto the surface of nanoparticles in a self-assembly manner [10]. The advantage of this route is the access of full predetermination and characterization of the polymer to ensure that the polymer shell fits the required applications. To achieve appropriate grafting densities and sufficient stabilization effects on metal-nanoparticle surfaces, strong interactions between the polymer and the surface are required. In this work, we use polyethylene with thiol or trithiocarbonate end-groups for the grafting-to approach. Both sulfur-containing moieties provide strong interactions with gold and silver surfaces [11,12,13]. 

We focus on polyethylene for its unique characteristics (mechanical, chemical, and thermal resistance), wide application, and low price. Until now, various composite materials from PE and different particles have been synthesized by in-situ polymerization, with is the immobilization of a catalyst onto a surface, or by blending nanoparticles into a polyethylene matrix [14,15,16,17,18]. Nevertheless, surface-modification with well-defined (low dispersity, high end-group functionalization) polyethylene is still challenging and only a few examples exist, such as the surface modification of PE on silica and iron oxide [19,20,21]. To the best of our knowledge, there is no protocol on the modification of gold/silver nanoparticles including polyethylene. To integrate the favored properties of PE and gold/silver nanoparticles simultaneously in a straight-forward manner, we applied an established technique for the synthesis of end-group functionalized polyethylene with a narrow mass distribution called catalyzed chain growth, which is based on a transition metal catalyst in combination with a chain transfer agent [22]. From this synthesis, various functional end-groups like hydroxy, amine, iodine, trithiocarbonate, thiol, or epoxides, are available for polyethylene [23,24,25]. 

In this study, by using thiol/trithiocarbonate end-groups, PE can effectively self-assemble onto the surface of gold/silver nanoparticles in solution, forming a metal-core−polymer-shell structure. Due to the PE shell, these nanohybrids can be fully dispersed in a PE matrix and show a completely reversible temperature-switchable solubility.

## 2. Materials and Methods

### 2.1. Materials

Ethylene (99.9%) and argon (99.999%) were purchased from Linde AG (Munich, Germany). Toluene (HPLC grade) was bought from Sigma-Aldrich Chemie GmbH (Munich, Germany), and degassed and stored over a molecular sieve. Butyloctylmagnesium (20 wt % in heptane) was purchased from Chemtura Europe GmbH (Frauenfeld, Switzerland). AgNO_3_ (Fluka, 98%, Schwerte, Germany), oleylamine (ACROS organics, Geel, Belgium, C-18 content 80–90%), tetra-*N*-octylammonium bromide (ABCR, 98%), hydrogen tetrachloroaurate trihydrate (ABCR, Karlsruhe, Germany, 99.9%), and NdCl_3_ (Fisher Scientific GmbH, Schwerte, Germany, 99.9%) were used as received. Tetrahydrofuran (Sigma-Aldrich, Munich, Germany) was distilled over CaH_2_ and stored over a molecular sieve. Phenylmethanethiol, lithium pentamethylcyclopentadienide (>98%) *N*,*N*-dimethylformamide (DMF) (p.A.), sodium citrate tribasic dihydrate (99%), methanol (99.8%), sodium borohydride (96%), chloroform (p.A.), potassium hydroxide, aliquat 336, tosyl chloride (>98%), and hydrazine monohydrate (98%) were purchased from Sigma-Aldrich and used as received. 

### 2.2. Synthesis

#### 2.2.1. Synthesis of (C_5_Me_5_)_2_NdCl_2_Li(OEt)_2_

The catalyst was synthesized according to the literature, by refluxing NdCl_3_ and lithium pentamethylcyclopentadiende in tetrahydrofuran for 12 h followed by removal of the solvent and extraction with diethylether [26].

#### 2.2.2. Synthesis of bis(benzylsulfinyl thiocarbonyl)disulfide

The synthesis was performed as described in the literature [27]. To a solution of tosyl chloride, aliquat 336 in dichloromethane a mixture of phenylmethanethiol, potassium hydroxide, aliquat 336, and carbon disulfide in water was added dropwise at −5 °C. The reaction mixture was extracted with dichloromethane. The organic phase was washed with aqueous NaHCO_3_ and water. The solvent was removed under vacuum. Recrystallization from acetone yielded bis(benzylsulfinyl thiocarbonyl)disulfide as yellow crystals.

#### 2.2.3. Synthesis of TOAB-Capped Gold Nanoparticles

Tetraoctylammonium bromid (TOAB) capped gold nanoparticles (AuNPs) were prepared by the Brust–Schiffrin method [28]. Briefly explained, hydrogen tetrachloroaurate trihydrate (94.5 mg, 0.240 mmol, dissolved in 20 mL nanopure water) and TOAB (524.9 mg, 0.960 mmol, dissolved in 20 mL toluene) were mixed to a round bottom flask and vigorously stirred for 30 min. The aqueous layer was removed and the organic layer was degassed with argon for 15 min. A freshly prepared solution of NaBH_4_ (72.6 mg, 1.92 mmol, in 5 mL water) was added dropwise under stirring. The mixture was stirred for a further 5 h. The organic layer was separated and washed with dilute sulphuric acid (5%, 10 mL), water (5 × 10 mL) and then dried over magnesium sulfate. The AuNPs prepared this way were used for functionalization reactions directly after their synthesis. The size distribution from the TEM analysis was added to the supplementary material (Figure S4).

#### 2.2.4. Synthesis of Silver Nanoparticles

Silver nanoparticles (AgNPs) were synthesized following a method reported elsewhere [29]. In a typical experiment AgNO_3_ (51 mg, 0.30 mmol) was dissolved in oleylamine (2.00 g, 7.48 mmol) with the aid of sonication. The oleylamine solution was then injected to 50 mL of refluxing toluene, which was purged with argon for 15 min. The solution was stirred for at least 16 h under argon. After the reaction, the mixture was cooled to room temperature and concentrated to ~15 mL via rotary evaporation. Afterwards, 160 mL of methanol was added to the mixture and the colloidal suspension was centrifuged at 956 g for 20 min and the supernatant was removed. The segregated material was redispersed by adding 12 mL hexane and precipitated again with 80 mL of methanol. The segregated material was gathered through centrifugation (27 g, 5 min). After an additional redisperse-centrifugation cycle (hexane/methanol) oleylamine-capped AgNPs were dispersed in toluene for further experiments. The size distribution from the TEM analysis was added to the supplementary material (Figure S5).

#### 2.2.5. Synthesis of Trithiocarbonate Terminated Polyethylene

In comparison to the literature [30], the polymerization of ethylene was performed in a 500 mL glass reactor at 80 °C. The reactor was filled with 400 mL dry toluene, degassed by a vacuum pump, and saturated with ethylene. The system was connected to an ethylene feedstock and the pressure was constant at 2.5 bar. Butyloctylmagnesium and the neodymium catalyst in toluene (ratio 200:1) were added and the reaction was carried out until precipitation was observed. At this point ethylene was removed and bis(benzylsulfinyl thiocarbonyl)disulfide (10 eq./[Mg]) in dry THF was added. After 8 h at 80 °C, the reaction mixture was cooled to room temperature and the polymer was precipitated in methanol, filtered and washed several times with methanol. The collected polymer was dried under vacuum at 50 °C for 18 h.

#### 2.2.6. Synthesis of Polyethylenethiol

Trithiocarbonate terminated polyethylene (1 eq.) and hydrazine monohydrate (20 eq.) was stirred at 90 °C for 20 h in a mixture of toluene and DMF (1:1). The reaction mixture was cooled to ambient temperature, the polymer was precipitated in methanol, filtered, washed several times with methanol, and dried overnight under vacuum at 50 °C.

#### 2.2.7. Preparation of Core-Shell Particles

Trithiocarbonate terminated polyethylene or thiol terminated polyethylene (2 mg) were dissolved in toluene (1 mL) at 90 °C. To the solution, silver- or gold-nanoparticles in toluene (2 mL, *c* = 2 mg/mL) were added and stirred for 30 min at 90 °C. The solution was used without purification for further usage.

### 2.3. Methods

#### 2.3.1. Transmission Electron Microscopy (TEM)

For TEM analysis a Philips CM 12 transmission electron microscope (Eindhoven, Netherlands), equipped with an Olympus CCD-camera (1376 × 1032 pixel) and 120 kV acceleration voltage, was applied. In the condenser lens, a 50 µm aperture was used, while scattered electrons were blocked with a 20 µm aperture. All samples were prepared by hot drop-casting (90 °C in toluene) on a Plano 200 mesh copper grid holding an amorphous carbon film. The grid was immediately covered with a glass cup and the solvent was slowly evaporated.

#### 2.3.2. High Temperature Size Exclusion Chromatography (HT-SEC)

HT-SEC characterizations were performed using an Agilent G1888 network headspace autosampler, an Agilent 1260 pump, an Agilent 1322A degasser and a PSS 246 interface. The system contained a polefin 10 µm precolumn and three polefin separation columns (10^3^ Å, 10^5^ Å, 10^6^ Å) and operated at 150 °C using 1,2,4-trichlorobenzene as eluent. A two channel Q 4 IR detector for CH_2_- and CH_3_-signals was used. All samples were dissolved in 1,2,4-trichlorobenzene (3 mg/mL) at 160 °C for 1 h before the measurement was performed.

#### 2.3.3. Nuclear Magnetic Resonance Spectroscopy (NMR)

^1^H NMR spectra were recorded on a Varian Unity 300 (Palo alto, CA, USA). Deuterated toluene was used as a solvent and chemical shifts (*δ* in ppm) were referenced to the residual of the deuterated toluene signal. The analysis was carried out at a temperature of 80 °C.

#### 2.3.4. Dynamic Light Scattering (DLS)

DLS was conducted on a Malvern Zetasizer Nano S instrument (Kassel, Germany), equipped with a He–Ne laser (*λ* = 633 nm). All samples were diluted in toluene, measured at 25 °C or heated to 90 °C and left for several hours prior to the measurements at 90 °C with a scattering angle of 173°.

#### 2.3.5. UV/visible Spectroscopy

UV/vis spectroscopy was performed with a Cary 300 scan photospectrometer (Santa Clara, CA, USA) in Hellma quartz cuvettes (path length: 10 mm). The spectra were recorded in a wavelength range of 250 to 800 nm with a scan rate of 150 nm/min at different temperatures (25 to 90 °C) in a baseline correction mode by subtracting the pure dispersant. 

## 3. Results and Discussion

### 3.1. Synthesis

**Scheme 1:**
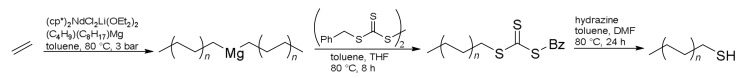
Applied synthesis route for the formation of functionalized polyethylene.

The modification of both silver- or gold-nanoparticles with polyethylene (trithiocabonate- or thiol group terminated) was performed by a grafting-to approach. The polymerization was conducted via catalyzed chain growth by an metallocene precatalyst ((cp*)_2_NdCl_2_Li(OEt_2_)_2_) in combination with a diorganomagnesium compound working simultaneously as an activator and chain transfer agent, resulting in magnesium end functionalized PE (Scheme 1). The trithiocarbonate end-group was introduced via a procedure described in the literature [30]. The yield of end-group functionalization was determined via ^1^H NMR spectroscopy by comparing the signals of the methylene-group in benzylic position and the methyl end-group of the polyethylene chain (supplementary Material Figure S1). The polyethylene chain possessed an end-group functionalization of 62%. We applied an aminolysis protocol for the conversion of a trithiocabonate group into a thiol group [31]. Polyethylenethiol was received quantitatively (functionalization: 62%, determined by ^1^H NMR, supplementary material Figure S2) after aminolysis of the trithiocarbonate group with hydrazine (24 h at 80 °C in toluene). The obtained polymer was analyzed by SEC. The chromatograph shows a strong shoulder (supplementary material Figure S3), resulting from differing elution times of end-functionalized and unfunctionalized PE. For this reason, molecular weight was estimated using the peak maximum (*M*_P_ = 2.5 × 10^3^ g mol^−1^). 

**Scheme 2:**
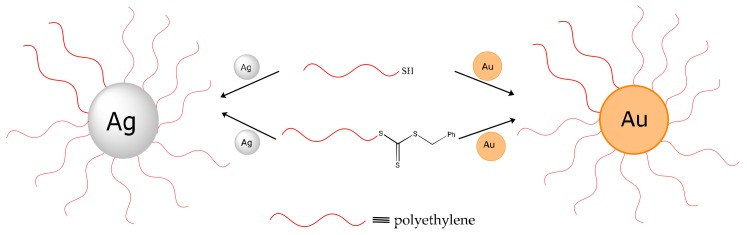
Synthesis of polyethylene functionalized silver and gold nanoparticles.

The end-group functionalized PE was employed for the formation of core–shell-structures with gold and silver nanoparticles. Due to the poor solubility of PE, the utilized silver and gold nanoparticles had to provide an approximately equivalent solubility in toluene. Based on this consideration, we chose tetraoctylammonium bromide (TOAB) capped gold from Brust–Schiffrin synthesis and oleylamine capped silver nanoparticles. Both TOAB and oleylamine are reported to bind weakly to the surface, enabling a ligand exchange with polymers containing sulfur end-groups [11]. It is necessary to mention that for oleylamine capped AgNP a certain purification process (several centrifugation cycles) is required to remove the excess of oleylamine and improve surface activity [11]. The attachment of the polymer to the highly active metal surface was carried out via coordination of trithiocarbonate- or thiol end-groups of the polymer. As in a typical experiment, end-group functionalized PE was dissolved in toluene at 90 °C. This high temperature is necessary to achieve complete solvation and a high flexibility of the polyethylene chains. Toluene dispersed nanoparticles were added to the polymer solution and the mixture was stirred for 30 min at 90 °C (Scheme 2). To avoid oxidation of the silver particles at higher temperatures, the functionalization of these particles was performed under an argon atmosphere.

### 3.2. Characterization of the Core–Shell Nanohybrids

Primal DLS, as well as TEM analysis showed that in both cases (thiol and trithiocarbonate end-group) the anchoring of respectively terminated PE on AuNPs and AgNPs surfaces was successful and the core–shell-particles were stable, even at higher temperatures. Both end groups showed different advantages: trithiocarbonate terminated polyethylene can act as a macro chain transfer agent in a RAFT-polymerization and therefore provides the possibility for the formation of block copolymers [24]. The thiol end-group is useful for further end-group modification using thiol-ene chemistry [32,33]. Due to the efficient synthesis of trithiocarbonate terminated PE, the following experiments and analyses focused on this species. 

**Figure 1:**
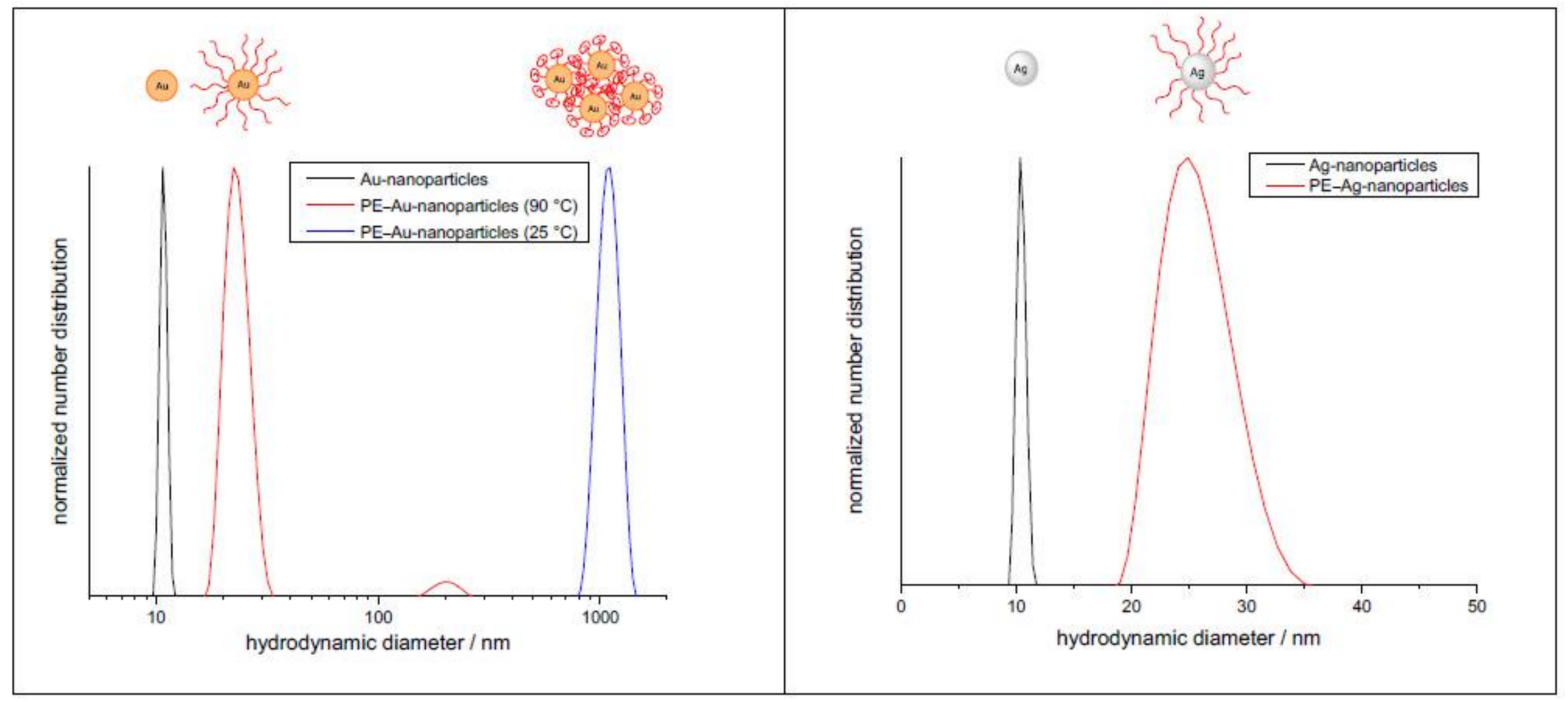
Number distributions from dynamic light scattering measurements of unfunctionalized and polyethylene-functionalized gold- and silver-nanoparticles. All samples were recorded in toluene at different temperatures and normalized to the peak maximum.

Using dynamic light scattering, the hydrodynamic diameters of unfunctionalized and polyethylene-capped nanoparticles were analyzed. The DLS experiments were carried out in toluene and performed at different temperatures. The unfunctionalized gold nanoparticles showed a size distribution with a maximum of ~10 nm (Figure 1, left), which was independent from the applied temperature. In contrast to this, the PE–gold-nanohybrids showed an interesting behavior. At 90 °C the curve of PE–Au-particles shifted towards higher hydrodynamic diameters (~22 nm), indicating the quantitative functionalization and high stability of core–shell-particles, even at high temperatures. After cooling, the hydrodynamic diameter of the PE capped AuNPs shifted to ~1000 nm, due to the aggregation of PE–AuNP. From these observations it can be extracted that the PE capped AuNPs have a similar solubility to PE. Below the critical solution temperature of polyethylene, the polymer shell becomes insoluble and the PE–Au-particles segregate from the solvent, ending in an aggregation of the particles. This temperature responding solubility is reversible. After reheating the sample to higher temperatures, a decrease in the hydrodynamic diameter was observed in the DLS experiment, confirming a complete and spontaneous redispersion of PE–AuNPs in toluene. Similar phenomena were also observed for PE–AgNPs. The distribution of polyethylene coated silver nanoparticles exhibited a strong increase in the hydrodynamic particle diameter, with a maximum at ~28 nm, whereas the untreated silver core reached maximum at ~11 nm (Figure 1, right). These results indicate that the polymer-shell exhibited an appropriate population density, a strong adhesion to the surface, and was thus entirely responsible for the solution properties of the particles.

**Figure 2:**
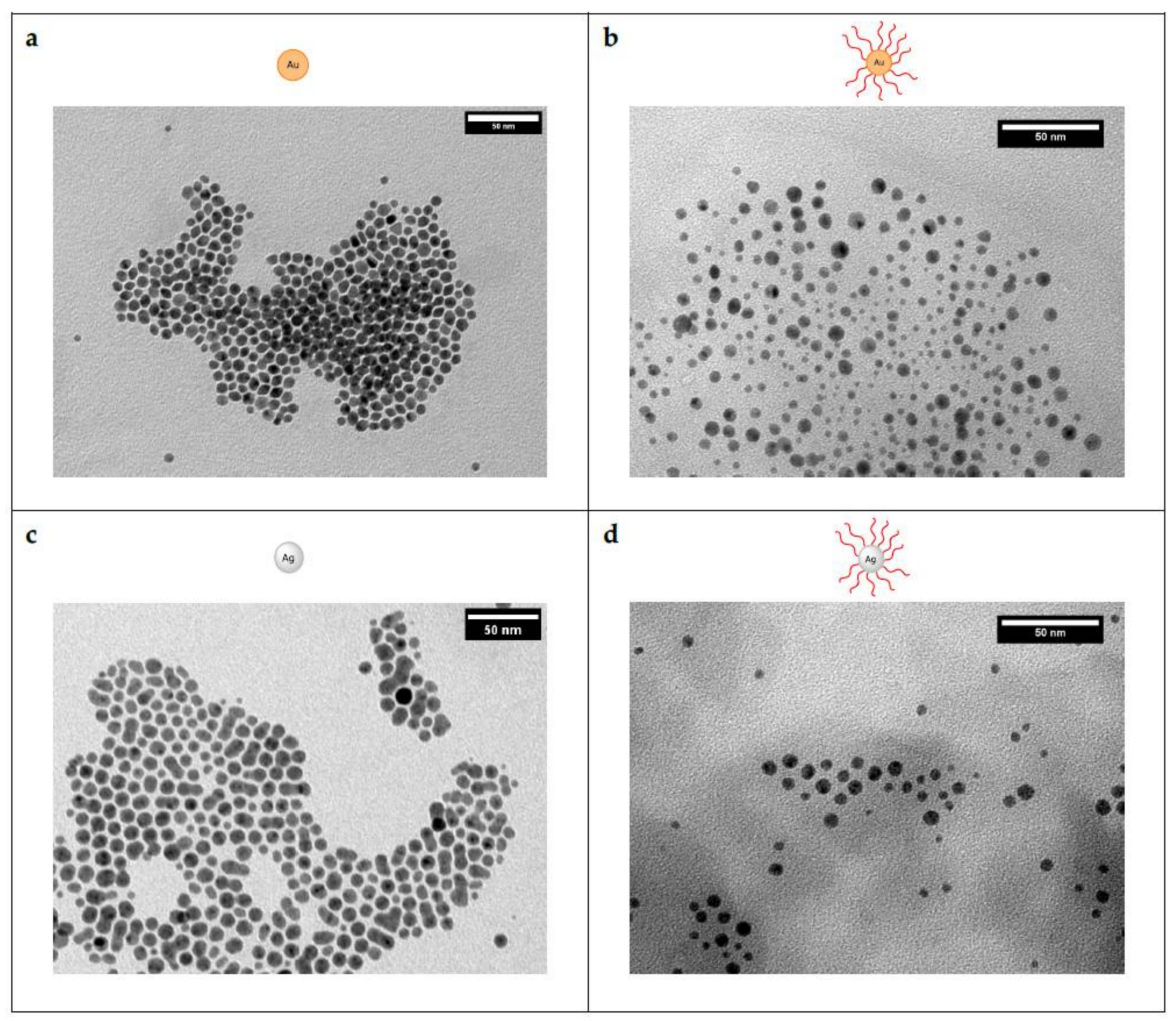
TE micrographs of unfunctionalized (**a**,**c**) and polyethylene grafted (**b**,**d**) gold and silver nanoparticles, respectively, showing an increase in interparticle distance.

In order to visualize and extend the data obtained from DLS analysis, transmission electron microscopy was performed, additionally. The TE micrographs of unfunctionalized gold, as well as silver nanoparticles show a strong tendency to aggregate in a solid state because of strong attractive interparticle forces (Figure 2). Comparing the TE micrograph of unfunctionalized Au/AgNPs with PE-Au/AgNPs, respectively (Figure 2), we observe a remarkable increase in inter-particle distance in PE-Au/AgNPs. Perfect separation and increased inter-particle distance indicates the effective formation of a PE shell on NPs from our grafting-to protocol. It is notable that the interparticle distance was remarkably high compared to the low molecular weight of this and other types of polymer [34]. One possible explanation is that linear HDPE with a high crystallinity (>90%) was used, resulting in a stretched form of the polymer. In the case of silver nanoparticles with narrower particle size distribution, we observed a hexagonal self-assembly pattern in some regions, as reported elsewhere [34].

### 3.3. Incorporation into a Polyethylene Matrix

**Figure 3:**
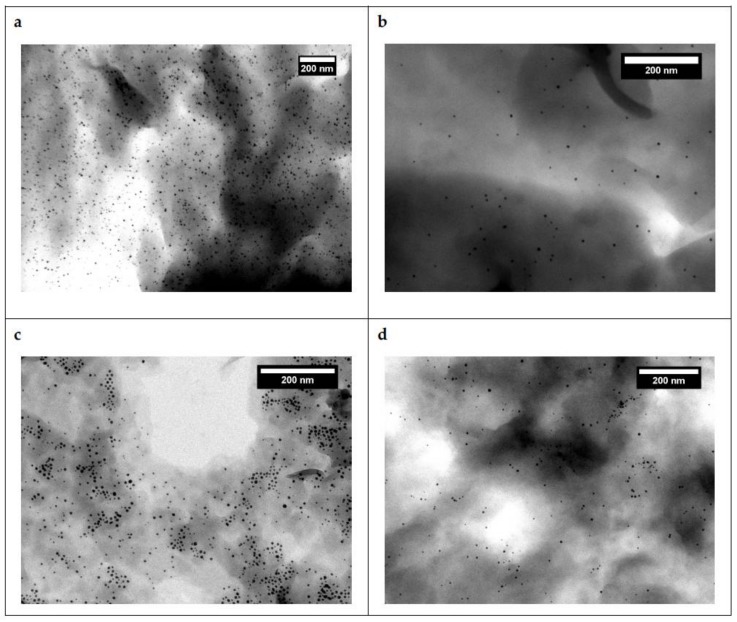
TE micrographs of dispersed PE–AuNPs with a high (**a**) and low (**b**) particle content and for PE–AgNPs with high (**c**) and low (**d**) particle content in a polyethylene matrix.

The perfect dispersing of gold and silver nanoparticles in PE matrices requires the appropriate surface properties of the respective nanoparticles. To demonstrate the advantage of introducing an analogous polymer shell, we investigated the compatibility of the capped nanoparticles with a polymer matrix by incorporating them into a commercially available HDPE (*M*_w_ = 7.2 × 10^3^ g·mol^−1^). The matrix polymer was dissolved in toluene at 90 °C and blended with PE–AuNPs or PE–AgNPs in two different particle dosages. These composite materials were characterized by TEM (Figure 3). The grafted AuNPs and AgNPs showed a complete dispersion in the polyethylene matrix at high (~10–15%, Figure 3a,c) and at low (~4%, Figure 3b,d) particle contents. In Figure 3c,d it can be observed that the particles are exclusively dispersed in the polyethylene matrix (grey shadow in the TE micrographs), whereas areas with no polymer are particle free. These results show a fine dispersion of the core–shell-particles within the matrix polymer, even at high particle/polymer ratios. 

PE capped gold nanoparticles possess excellent long-time stability due to their dense polymer shell, as described previously for other core–shell-particles [35]. For verification, polyethylene–gold and ungrafted particles were dissolved in toluene, followed by evaporation of the solvent. The core–shell particles were stable over a month and were completely redispersible in toluene at 90 °C. In contrast, the unfunctionalized AuNPs showed a strong tendency toward irreversible aggregation and were therefore not redispersible, even with the aid of ultrasonication. The plasmonic color of AuNPs is very useful to check the aggregate state of AuNPs [3]. In a solid state, the PE–Au-particles keep their red color, indicating good dispersion. The ungrafted Au-particles change color from red to black, showing the occurrence of aggregation, and even under ultrasonic treatment, the sample with unfunctionalized AuNPs remains a blue color. The aggregating of unfunctionalized AuNPs has also been studied with UV/vis spectroscopy [36]. We observed a strong red-shift of the absorption maximum of AuNPs from 530 nm for fresh prepared AuNPs, to 546 nm for aged unfunctionalized samples, while no observable red-shift from PE–AuNPs was observed (supplementary material Figure S6). The effective capping of PE on AuNPs can therefore enhance the stability of nanocomposites significantly. As a protecting shell, polyethylene has an advantage over other polymer due to its strong chemical and mechanical stability and durability [37].

### 3.4. Thermoresponsive Behavior of the Cores–Shell-Nanoparticles

**Scheme 3:**
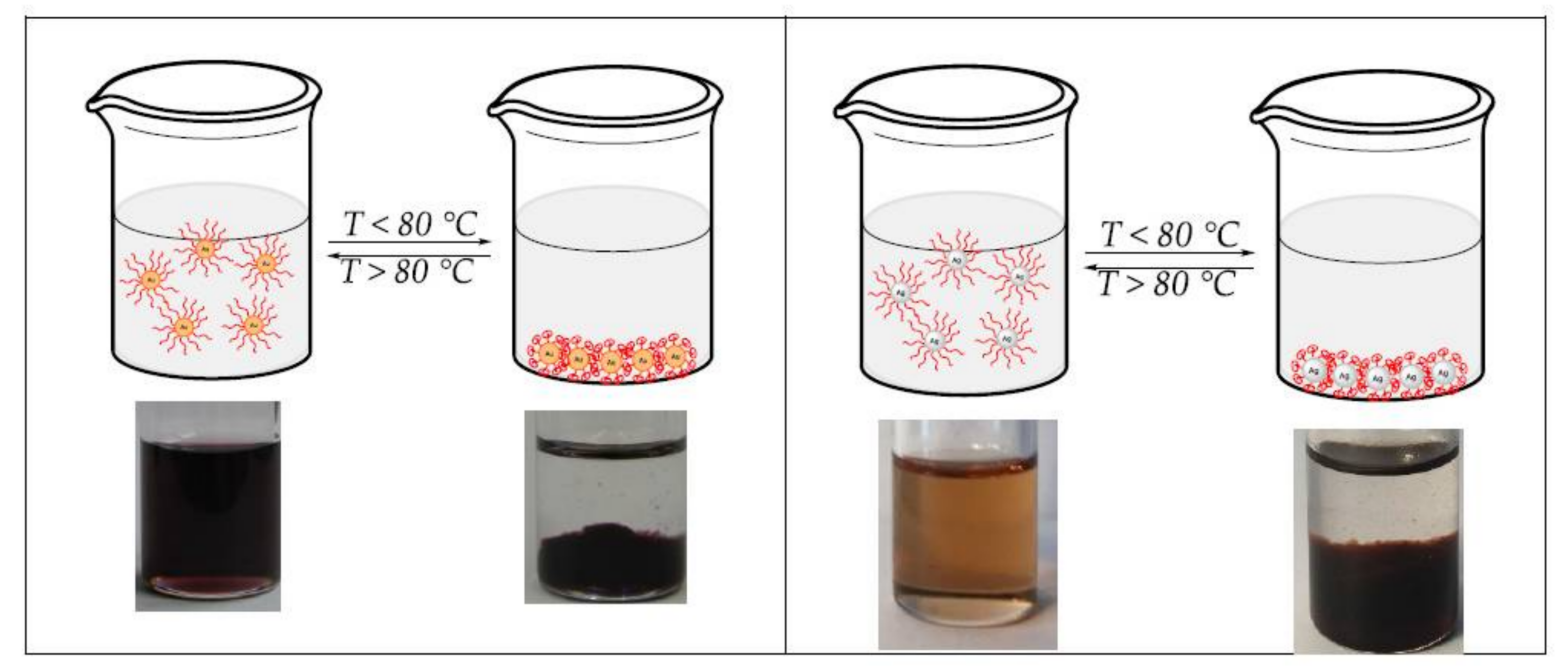
Temperature dependent solubility of polyethylene grafted gold and silver nanoparticles.

The thermoresponsive solubility of polyethylene grafted nanoparticles is of great interest, as reported for polyethylene supported homogenous catalysts [38,39,40]. Due to the strong temperature dependent solubility of polyethylene, an easy and instant separation/regeneration of polyethylene supported materials is accessible [41]. The quick, switchable solubility of our PE grafted AuNPs and AgNPs could be observed directly by the naked eye (Scheme 3). Above the solution temperature of the polyethylene shell, the nanocomposites remained a well-dispersed homogenous sol. At lower temperatures, segregation of the polymer and the solvent occurred and therefore the mixture formed, quantitatively, a two-phase system of aggregated particles and completely colorless, particle-free solvent. This spontaneous separation required no centrifugation or other treatment. After reheating the samples, the dispersion proceeded spontaneously. The use of polyethylene as a polymer shell enhances the field of the temperature-dependent solubility of gold particles compared to other stabilized particles, as described previously [41,42].

**Figure 4:**
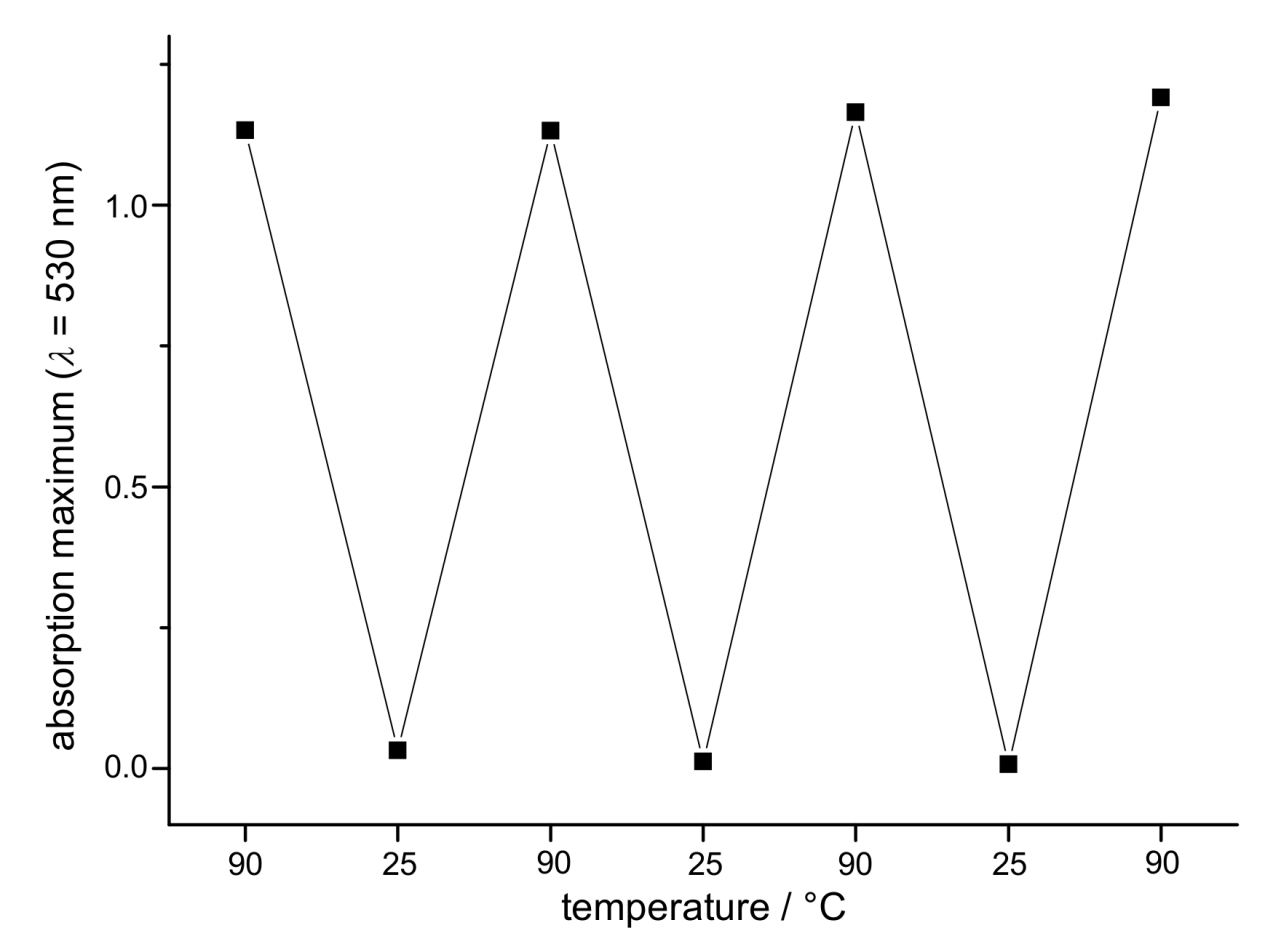
UV–vis measurement for temperature cycling on PE-AuNPs in toluene. The low value of absorption at 25 °C indicates the absence of PE-AuNPs in the light pathway, which is located in the middle of cuvette. In this case, PE-AuNPs are segregated, as showed in Figure 1.

To confirm the reversibility of the temperature responding behavior, UV/vis spectroscopy was applied to analyze the gold particles in toluene. We tracked the maximum absorption (*λ* = 530 nm) for four temperature cycling. After tempering at 90 °C (12 h), the sample was cooled down and allowed to rest at 25 °C for 12 h before the UV/vis measurement was repeated (Figure 4). The absorbance maximum of the dissolved core–shell nanoparticle sample at 90 °C showed a value around 1.1; whereas, at 25 °C no appreciable absorbance was observed. Four cycles were repeated showing a perfect reversal of temperature switchable solubility, without any shifting of the maxima. This result indicates that gold nanoparticles can transfer between both sediment and dispersed state without having structural damage as described in Scheme 3. As a double-check measurement, unmodified gold nanoparticles were blended into a polyethylene matrix. In this case, the described segregation was not observed.

**Figure 5:**
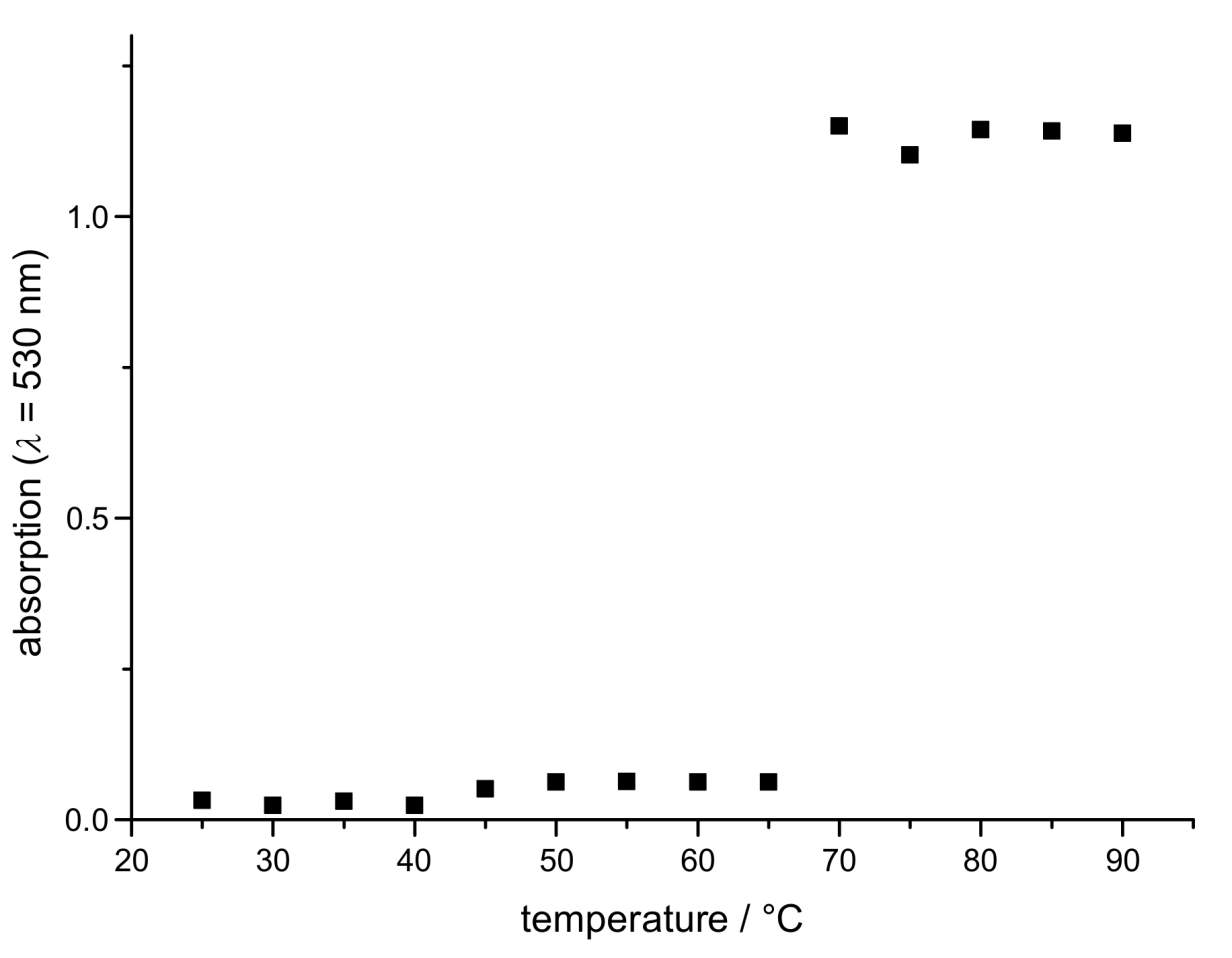
Temperature dependent UV/vis measurement of polyethylene-gold-nanoparticles in toluene at *λ* = 530 nm.

We studied the temperature dependent dispersibility of PE-AuNPs in toluene with a step-by-step heating procedure monitored by UV–vis spectroscopy. To investigate the critical solution temperature of PE-AuNPs, the absorbance at *λ* = 530 nm and at different temperatures was recorded (Figure 5). The temperature was raised in 5 °C steps, with a waiting period of 2 h between each measurement. Between 25 and 65 °C, no significant increase in absorbance was observed, indicating that no dispersion of the PE-AuNPs occurred below 65 °C. Above 70 °C, the PE shell reached its “solution temperature” while still anchored on the AuNPs, making the PE-AuNPs dispersible as a homogenous colloid, which was detected by a strong increase in absorbance. It has to be mentioned that at 70 °C the absorbance maxima increased over 2 h to its maximum value; whereas, at 90 °C only 10 min were required for a complete solution of the capped nanoparticles.

## 4. Conclusions

In this article, we presented a straight-forward and effective synthesis of PE modified AuNPs and AgNPs using a grafting-to approach. We introduced a strong anchoring ability of PE on AuNPs and AgNPs by designing a well-defined trithiocarbonate or thiol terminated polyethylene through a catalyzed chain growth route. The formation of PE shells on AuNPs and AgNPs was confirmed by both TEM and DLS. PE-Au/AgNPs was shown to be perfectly dispersed within a polyethylene matrix at different ratios. PE capped nanoparticles have proven to be very robust over long temperature ranges and times, especially compared to unfunctionalized particles. Furthermore, the PE capped Au-nanoparticles exhibited a completely reversible, temperature switchable dispersibility in toluene, enabling a complete separation/regeneration of the colloid particles from the solvent.

## Supplementary Materials

The following are available online at http://www.mdpi.com/2073-4360/10/4/407/s1. ^1^H NMR spectra of trithiocarbonate- and thiol-terminated polyethylene. Size exclusion chromatogram of trithiocarbonate PE, histogram of AuNP and AgNP, UV/vis spectra of aged AuNP and PE–AuNP. Figure S1: ^1^H NMR of trithiocarbonate terminated polyethylene, Figure S2: ^1^H NMR of thiol terminated polyethylene, Figure S3: Size exclusion chromatogram of trithiocarbonate terminated polyethylene, Figure S4: Size distribution of the applied AuNP, Figure S5: Size distribution of the applied AgNP, Figure S6: UV spectra of unfunctionalized and PE grafted gold nanoparticles after aging.
