# Supplementary material for: Polyethylene-Grafted Gold and Silver Nanoparticles Using Catalyzed Chain Growth (CCG)
Source: Polymers (Basel). 2018 Apr 6;10(4):407. doi: 10.3390/polym10040407 (PMC6415259; doi:10.3390/polym10040407)
Supplement: Supplementary file 1 [file polymers-10-00407-s001.pdf]

polymers

Supplementary Material

Polyethylene covered gold and silver nanoparticles using Catalyzed Chain Growth (CCG)

Jannik Wagner, Wentao Peng and Philipp Vana*

Institute of Physical Chemistry, Georg-August-University Göttingen, Tammannstr. 6, D-37077 Göttingen, Germany

* Correspondence: pvana@uni-goettingen.de; Tel.: +49-(0)551-39-12753

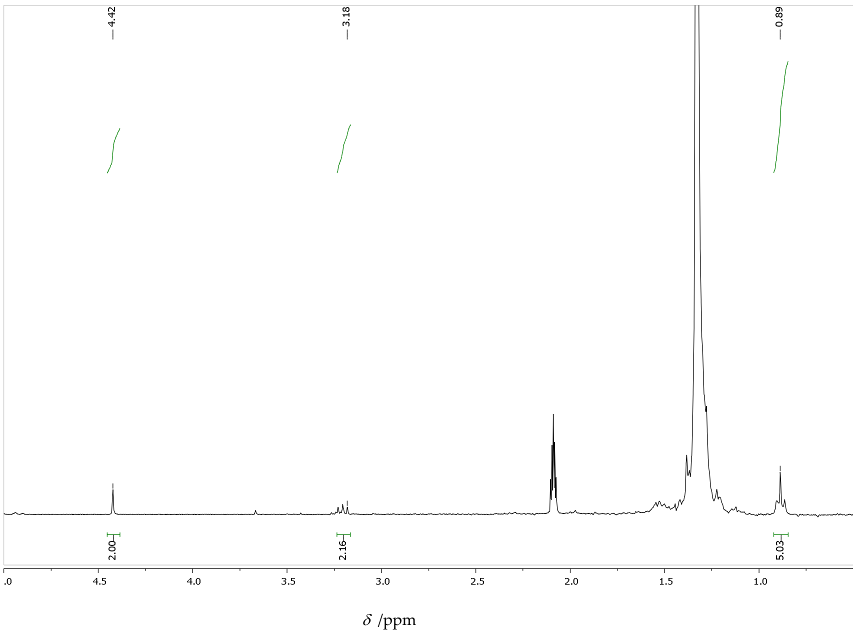

Figure 1: ^{1}H-NMR of trithiocarbonate terminated polyethylene. The measurement was conducted in toluene at 80 °C.

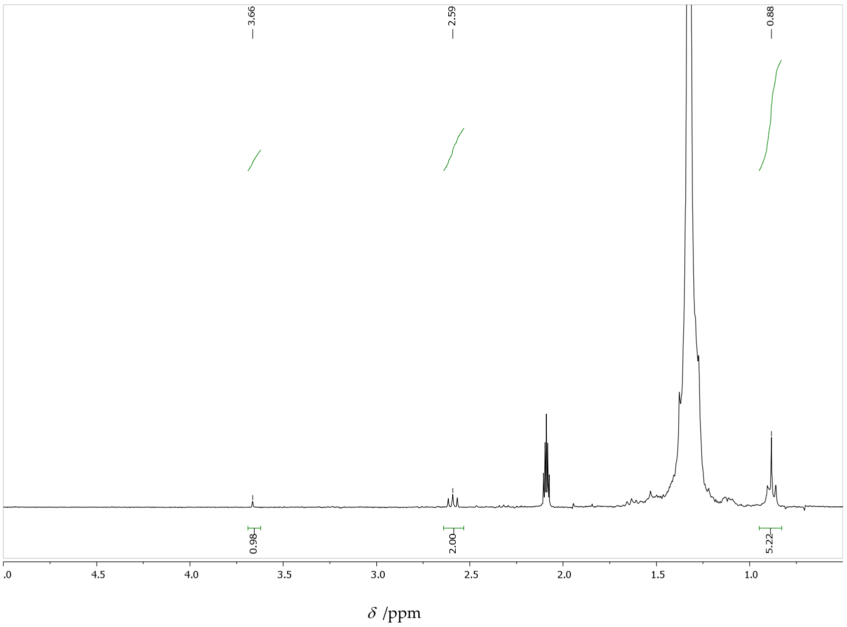

Figure 2: ^{1}H-NMR of thiol terminated polyethylene. The measurement was conducted in toluene at 80 °C.

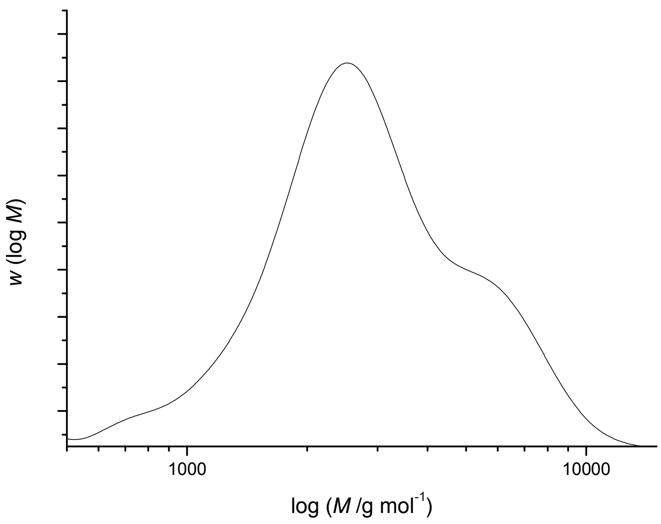

Figure 3: Size exclusion chromatogram of trithiocarbonate terminated polyethylene.

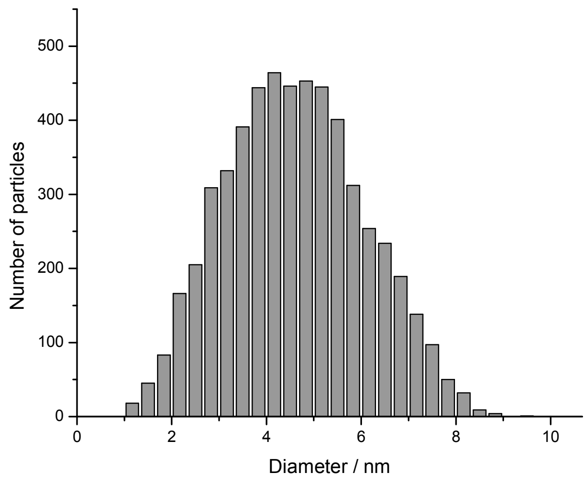

Figure 4: Size distribution of the applied AuNP.

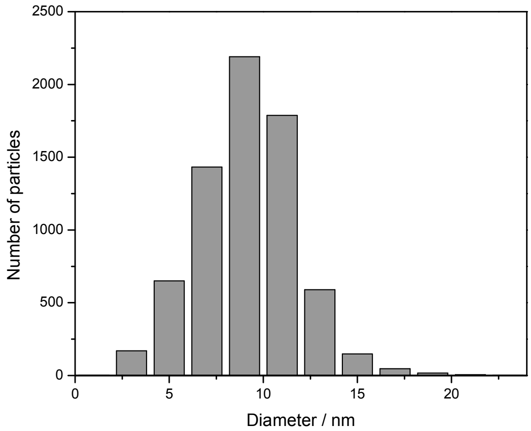

Figure 5: Size distribution of the applied AgNP.

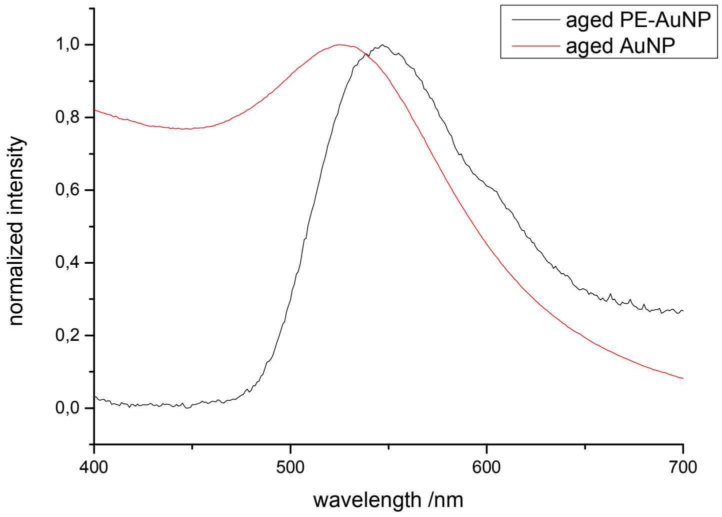

Figure 6: UV spectra of unfunctionalized and PE grafted gold nanoparticles after aging.
